# An alignment-free heuristic for fast sequence comparisons with applications to phylogeny reconstruction

**DOI:** 10.1186/s12859-020-03738-5

**Published:** 2020-11-18

**Authors:** Sriram P. Chockalingam, Jodh Pannu, Sahar Hooshmand, Sharma V. Thankachan, Srinivas Aluru

**Affiliations:** 1 Department of Computer Science, University of Central Florida, 4000 Central Florida Blvd, Orlando, USA; 2 Institute for Data Engineering and Science, Georiga Institute of Technology, 756 W Peachtree Street NW, Atlanta, USA; 3 Department of Computational Science and Engineering, Georiga Institute of Technology, 756 W Peachtree Street NW, Atlanta, USA

**Keywords:** Alignment-free methods, Sequence comparison, Phylogeny reconstruction

## Abstract

**Background:**

Alignment-free methods for sequence comparisons have become popular in many bioinformatics applications, specifically in the estimation of sequence similarity measures to construct phylogenetic trees. Recently, the *average common substring* measure, *ACS*, and its *k*-mismatch counterpart, *ACS*_*k*_, have been shown to produce results as effective as multiple-sequence alignment based methods for reconstruction of phylogeny trees. Since computing *ACS*_*k*_ takes *O*(*n* log*kn*) time and hence impractical for large datasets, multiple heuristics that can approximate *ACS*_*k*_ have been introduced.

**Results:**

In this paper, we present a novel linear-time heuristic to approximate *ACS*_*k*_, which is faster than computing the exact *ACS*_*k*_ while being closer to the exact *ACS*_*k*_ values compared to previously published linear-time greedy heuristics. Using four real datasets, containing both DNA and protein sequences, we evaluate our algorithm in terms of accuracy, runtime and demonstrate its applicability for phylogeny reconstruction. Our algorithm provides better accuracy than previously published heuristic methods, while being comparable in its applications to phylogeny reconstruction.

**Conclusions:**

Our method produces a better approximation for *ACS*_*k*_ and is applicable for the alignment-free comparison of biological sequences at highly competitive speed. The algorithm is implemented in Rust programming language and the source code is available at https://github.com/srirampc/adyar-rs.

## Background

Over the past two decades, many similarity measures based on alignment-free methods have been proposed for sequence comparison for a diverse range of bioinformatics applications. With the increasing availability of sequence data from multiple sources and as alignment algorithms are reaching their limits, many of these alignment-free methods have become popular in applications such as phylogeny reconstruction, sequence clustering, transcript quantification and detection of horizontal gene transfers [1, 2].

For phylogeny reconstruction, alignment-free methods are used to construct the pairwise distance matrix, a symmetric matrix of sequence similarity measures computed for every pair in the given set of sequences. With the distance matrix as their input, algorithms such as unweighted pair group method with arithmetic mean (UPGMA) [3] or neighbor-joining (NJ) [4] construct the desired tree.

Alignment-free methods for computation of similarity measures can be classified based on whether the seeds are exact or approximate and whether the seeds are of fixed- or variable-length. The most popular among the fixed-length exact seed methods are *k*mer-based techniques, which proceed by first constructing the sets of all the *k*mers (*k*mers are fixed-length exact seeds of length *k*) of a pair of sequences, followed by the estimation of a similarity measure either based on the *k*mer frequency profile (Eg. Euclidean distance, CVTree [5],FFP [6]) or based on the intersection/differences of the *k*mer sets (Eg. Jaccard coefficient). Lu et al. [7] presents a comprehensive review of about 28 different such measures typically used in the construction of phylogeny trees. Methods using approximate fixed-length such as spaced-seeds approaches [8] allow the use of *k*mers with mismatches at specific locations and make use of multiple patterns to improve accuracy.

Among the variable-length seeding methods, one of the measures shown to be effective in phylogeny applications is the *average common substring*, *ACS*, which is computed for a pair of sequences as the mean of the lengths of the longest common prefixes [9]. After computing the *ACS*, the sequence similarity of two sequences X and Y is computed as follows:

1 $$ d(\mathsf{X}, \mathsf{Y}) = \frac{1}{2} \left(\frac{\log |\mathsf{Y}|}{\mathsf{ACS}(\mathsf{X}, \mathsf{Y})} + \frac{\log |\mathsf{X}|}{\mathsf{ACS}(\mathsf{Y}, \mathsf{X})} \right) - \left(\frac{\log |\mathsf{X}|}{|\mathsf{X}|} + \frac{\log |\mathsf{Y}|}{|\mathsf{Y}|} \right)   $$

By introducing *k* mismatches into the *average common substring* metric (abbreviated as *ACS*_*k*_), Leimeister and Morgenstern [10] demonstrated improved accuracy for phylogeny applications. However, their approach, called *kmacs*, uses a greedy heuristic as an approximation of *ACS*_*k*_ since computing exact *ACS*_*k*_ is computationally expensive and was shown to take *O*(*n* log*kn*) for a pair of sequences of total length *n* [11]. In a later work, Thankachan et. al. [12] also proved that the runtime bounds remain *O*(*n* log*kn*) even when insertions and deletions allowed along with mismatches. Based on [11, 13] presented another greedy heuristic to approximate *ACS*_*k*_.

In this work, we present a novel linear-time heuristic that is a more accurate approximation of *ACS*_*k*_ than *kmacs*’ approach. While *kmacs* constructs an *ACS*_*k*_ approximation by means of a forward extension of the longest common prefixes, our algorithm performs both forward and backward extensions to identify a *k*-mismatch common substring of longer length, and hence, producing a closer approximation to the exact *ACS*_*k*_. Using three real datasets, we evaluate the runtime, accuracy and the effectiveness of our proposed approach. We also demonstrate its applicability for phylogeny tree construction.

## Methods

### Notations and preliminaries

Let X and Y be two sequences drawn from the alphabet set *Σ*. We denote the length of X by |X|, the suffix of X starting at the position *i* as X_*i*_. Also, we use $\overleftarrow {\mathsf {X}}$ and $\overleftarrow {\mathsf {Y}}$ to denote the reverse of the strings X and Y respectively.

Let |X|+|Y|=*n*. We define *LCP*(X_*i*_,Y_*j*_) to be the longest common prefix of X_*i*_ that matches with Y_*j*_ and *LCP*_*k*_(X_*i*_,Y_*j*_) its *k*-mismatch counterpart i.e., a longest common prefix that allows *k* mismatches, *k*≥0 (also termed as the longest *k*-mismatch substring starting at X_*i*_). We denote max*j*|*LCP*_*k*_(X_*i*_,Y_*j*_)| by *λ*_*k*_(*i*) and the position in Y corresponding to the *λ*_*k*_(*i*)-length match as *μ*_*k*_(*i*) i.e.,

$$\mu_{k}(i) = \arg \max_{j} \left|\mathsf{LCP}_k \left(X_{i}, Y_{j}\right)\right|, k \geq 0. $$

For the sake of brevity, we abbreviate *λ*_0_(*i*) and *μ*_0_(*i*) as *λ*(*i*) and *μ*(*i*) respectively.

The *average common substring*, *ACS* of X w.r.t. Y is defined as

2 $$ \mathsf{ACS}\left(\mathsf{X}, \mathsf{Y}\right) = \frac{1}{|\mathsf{X}|}\sum_{i=1}^{|\mathsf{X}|} \max_{j} \left|\mathsf{LCP}\left(\mathsf{X}_{i}, \mathsf{Y}_{j}\right)\right|.  $$

*ACS*_*k*_(X,Y),*k*≥0 of X w.r.t. Y is defined similarly with *LCP*_*k*_ instead of *LCP* in the above equation. Note that *ACS*_*k*_(X,Y)≠*ACS*_*k*_(Y,X).

We use *GST*_*f*_ and *GST*_*r*_ to denote the generalized suffix tree constructed for the concatenated strings $\mathsf {T} = \mathsf {X}\$_{1}\mathsf {Y}\$_{2} \text {and} \overleftarrow {\mathsf {T}} = \overleftarrow {\mathsf {X}}\$_{1}\overleftarrow {\mathsf {Y}}\$_{2}$ respectively, where *$*_1_,*$*_2_∉*Σ*. For our algorithm, *GST*_*f*_ and *GST*_*r*_ serve as an indexing data structures that enable us to perform longest common prefix queries for X and Y in constant time. Both *GST*_*f*_ and *GST*_*r*_ can be constructed in *O*(*n*) time with *O*(*n*) space.

### Previous greedy heuristics

Using the notations described above, *ACS*_*k*_(X,Y) is computed as $\mathsf {ACS}_{k}(\mathsf {X}, \mathsf {Y}) = \sum _{i=1} \lambda _{k}(i) / |\mathsf {X}|$. The key difficulty in computing *ACS*_*k*_ is the estimation of the array *λ*_*k*_(*i*),*i*=1,…,|X|. Before we present our linear-time approximate algorithm for computing *λ*_*k*_, we briefly discuss the previously established heuristic methods for approximating *λ*_*k*_.

In *kmacs* [10], the previously published greedy approach, *λ*_*k*_(*i*) is approximated by extending the longest common prefixes. *kmacs* uses the longest common substring of suffixes X_*i*_ and Y_*q*_,*q*= arg max*j*|*LCP*(X_*i*_,Y_*j*_)| as the initial anchor segment, then performs a forward extension to identify the common substring with *k*−1 mismatches and approximates the total length as *λ*_*k*_(*i*). For example, if X and Y are the strings CATTGCATACGA and ATGGATCCAATAG respectively, then to compute an approximation to *λ*_2_(4), *kmacs* would first identify the *LCP* match of X_4_ at Y_2_ and then approximate *λ*_2_(4) as 6 by matching the segments TGCATA and TGGATC. Formally, *kmacs* computes the following measure as an approximation of *λ*_*k*_(*i*):

$$\lambda(i) + 1 + \left|\mathsf{LCP}_{k-1} \left(\mathsf{X}_{i + \lambda(i) + 1}, \mathsf{Y}_{\mu(i) + \lambda(i) + 1}\right)\right|. $$

Using a generalized suffix tree constructed for X and Y, the above measure can be calculated in *O*(*k*) time via *k* consecutive *LCP* queries starting with X_*i*_ and Y_*μ*(*i*)_. Therefore, the similarity metric based on the above heuristic measure can be computed in *O*(*nk*) time.

*ALFRED-G* [13] follows a similar logic except that it includes an extra mismatch in the initial anchor segment. Formally, *ALFRED-G* approximates *λ*_*k*_(*i*) with the following measure:

$$\lambda_{1}(i) + 1 + \left|\mathsf{LCP}_{k-2} \left(\mathsf{X}_{i + \lambda_{1}(i) + 1}, \mathsf{Y}_{\mu_{1}(i) + \lambda_{1}(i) + 1}\right)\right|. $$

### Proposed algorithm

In our algorithm, we make use of the following observation: a *k*-mismatch common substring of two suffixes X_*i*_ and Y_*j*_ includes *k*−1 common substrings separated by *k* mismatch characters. This observation leads to the following key intuition behind our algorithm – the anchor segment can be any one of the *k*−1 segments that constitute a *k*-mismatch common string. As mentioned above, both *kmacs* and *ALFRED-G* consider the first segment as the anchor segment. Our heuristic, denoted by *λk*′(*i*), is computed by extending all *k*−1 matching substrings that overlap the position *i*, as anchors.

We illustrate our approach with the following example. Consider the three suffixes X_*p*_=AATCGGT...,Y_*q*_=AATGGGA... and Y_*r*_=AACCGGT..., and let *μ*(*p*)=*q*;*μ*(*p*+3)=*r*+3. A greedy heuristic based algorithm such as *kmacs*, which uses the *LCP* to find anchor segments, will select Y_*q*_ as the anchor point and approximate *λ*_1_(*p*) as 5, even though there is a better match at Y_*r*_. Extending the segments backward overcomes this limitation in this example because a backward extension from the Y_*r*+3_=CGG... segment from X_*p*+3_ can identify Y_*r*_ to be the better match for X_*p*_.

Algorithm 1 presents the pseudo-code for our proposed heuristic. It takes as input strings X and Y, and outputs an array *λk*′ of length |X|, whose *i*th entry contains the approximation for *λ*_*k*_(*i*).


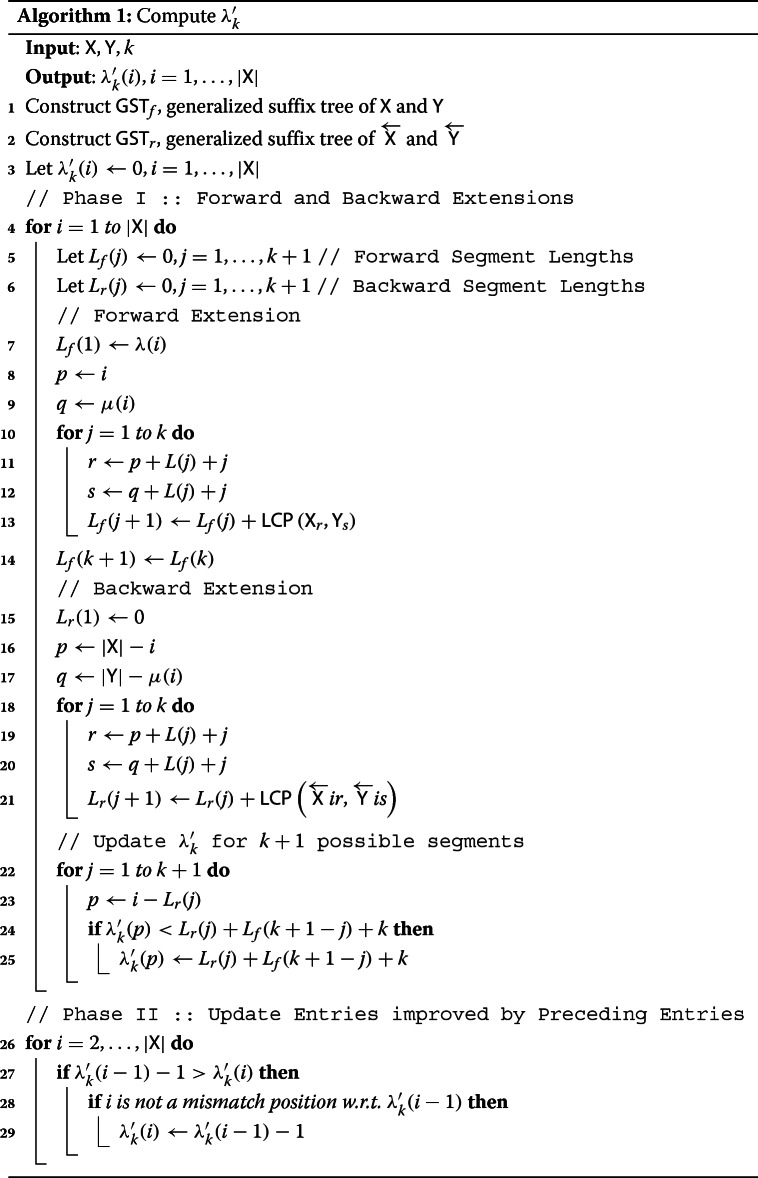


After constructing the two generalized suffix trees *GST*_*f*_ and *GST*_*r*_ and initializing the *λk*′(*i*) entries (Lines 1– 3), the algorithm proceeds in two phases. In the first phase, we compute the forward and backward extensions of the longest common substring for each position in X (Lines 4– 25). Here, we make use of two arrays *L*_*f*_ and *L*_*r*_, each of length *k*+1, which contain the lengths of the 0,1,2…,*k*-mismatch substrings starting and ending at position *i* respectively. *L*_*f*_ and *L*_*r*_ can be computed via *k* *LCP* queries on *GST*_*f*_ and *GST*_*r*_ respectively (Lines 13 and 21). After computing the *L*_*f*_ and *L*_*r*_ arrays, we update *λk*′ arrays for the *k*+1 possible positions corresponding to all the possible forward and backward extensions(Lines 22– 25).

In some cases, the approximation computed at position *i* in the first phase can be improved by examining the *λk*′(*i*−1) entry, if the *i*the position doesn’t correspond to a mismatch character of the preceding entry. In the second phase, we update those entries for whom a better approximation is one less than the preceding entry in *λk*′ (Lines 26– 29).

Since *LCP* queries take constant time using *GST*s, the first phase can be accomplished in *O*(*nk*) time and *O*(*n*+*k*) space. Since the second phase is just a left to right pass over the *λk*′ array and since the construction of the suffix trees also take *O*(*n*) time and space, our algorithm takes linear time.

### Implementation details

We implemented our algorithm in the Rust progamming language [14] and used the libdivsufsort library [15] to construct the suffix array data structures.

Our algorithm requires only the computation of *LCP* queries, which can be done only using generalized suffix arrays along with the longest-common-prefix (LCP) arrays and range minimum query (RMQ) data structures. The use of suffix and LCP arrays instead of the suffix trees significantly reduces the memory footprint for our implementation.

Whenever there are multiple options to extend the *LCP* match (i.e., there are multiple locations in which a common substring can be equal in length and be the longest), we apply the heuristic discussed earlier in this section to all possible locations and select the longest among them. In the worst case this can make the implementation to take *O*(*n*^2^*k*) time but in practice, it takes *O*(*nkz*) time, where *z* is the average number of maximal matches to a substring in Y starting at a position *i* in X.

The core computation of the algorithm demands multiple *LCP* queries. However, we observed that in most cases, the time taken to walk through the text to identify is faster compared to evaluating the *LCP* query. This is because of two reasons (a) modern CPU architectures include multiple hierarchies of caches, that enable faster access to data elements that are located with in relatively close storage locations and (b) most practical datasets have a distribution of relatively short *LCP* lengths. Therefore, in all our experiments reported in the next section, except for the case when *LCP* is 0, we walk the text to identify the longest common prefixes. When the *LCP* is 0 i.e., the case when there is no suffix match in Y for a suffix X_*i*_ or vice-versa, we estimate the *k*-mismatch *LCP* by the approximation computed for the suffix X_*i*+1_.

## Results and discussion

All the experiments were run on a system having two 2.4 GHz 14-Core Intel E5-2680 V4 processors and 256 GB of main memory, and running RedHat Enterprise Linux (RHEL) 7.0 operating system. Along with our implementation, we also ran *kmacs* [10] and *ALFRED-G* [13] for comparison. *kmacs* and *ALFRED-G* were compiled using gcc compiler version 8.3.0. Our implementation was compiled using rust compiler version 1.3.6.

To evaluate the runtime, the relative accuracy and the effectiveness of our proposed algorithm, we used four real datasets – *Primates*, *Roseobacter*, *BAliBASE* and *E. coli*, all of which have been previously used to evaluate alignment-free techniques to estimate sequence similarity [10, 13, 16].

*Primates* is a DNA sequence dataset collected from prokaryotic organisms and has 27 primate mitochondrial genomes with a total length of ≈450 kilobases. The reference phylogeny tree for this dataset was constructed based on multiple sequence alignment the 27 sequences.

*Roseobacter* dataset is a set of eukaryotic DNA sequences with a total length of ≈875 kilobases, collected from the coding regions of 32 Roseobacter genomes as described in [13]. *BAliBASE* dataset is a collection of 218 protien sequence datasets of total length ≈2.5 megabases, gathered from the BAliBASE V3.0 [17], a popular benchmark for evaluating multiple sequence alignment algorithms.

*E. coli* dataset is a collection of 29 whole genomes of E. coli/Shigella strains, originally compiled by [16]. This dataset has a total length of 138 megabases in which the size of the seqeunces range from 4.3 megabases to 5.4 megabases.

For the *Roseobacter* dataset, we used the phylogenetic tree presented in [18] as the reference tree. In case of *BAliBASE* datasets, the reference trees are constructed from the corresponding reference alignments using the *proml* program available in *PHYLIP* [19], which implements the Maxmimum Likelihood method. For the *E. coli* datasets, we used the phylogenetic tree presented in [16] as the reference tree.

We conducted experiments on all the software to evaluate (i) the accuracy of the estimated *ACS*_*k*_, (ii) runtime characteristics, and (iii) applicability of our algorithm to phylogeny reconstruction.

**Fig. 1:**
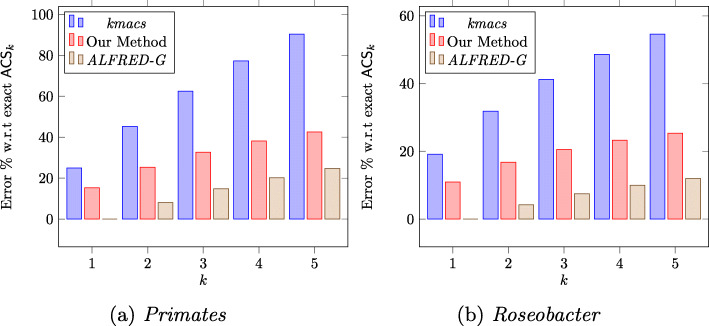
Avg. error percentage of estimated *ACS*_*k*_ w.r.t. exact *ACS*_*k*_

To estimate the accuracy of the *ACS*_*k*_ estimated using our heuristic, we approximate *ACS*_*k*_, for every pair of input sequence in the *Primates* and *Roseobacter* datasets, using our proposed heuristic with *k*=1,…,5. We, then, used the ALFRED software published by [20] to find the true value of *ACS*_*k*_, computed the error percentage of estimated *ACS*_*k*_ compared to the true values, and finally plotted the average error percentages against increasing values of *k*. Figures 1a and b illustrate the average deviation of the approximate values computed by the respective software from the exact value of *ACS*_*k*_ for *Primates* and *Roseobacter* datasets respectively.

Figures 1a and b show that the error percentage for our proposed method is less than that of *kmacs* in all the cases. Specifically, in case of *kmacs*, the error percentages can be as high as 80% with *k*=4 for the *Primates* datasets, where as our method shows a deviation of less than 40%. Even though the error percentages increases as *k* increases for both *kmacs* and our algorithm, compared to *kmacs*, the error rate grows relatively slower with increasing *k* for our method.

**Fig. 2:**
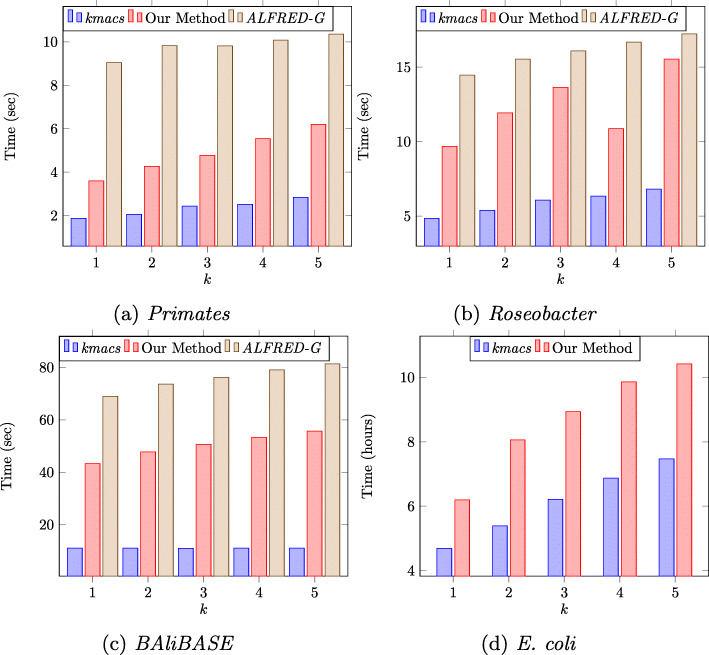
Total runtime of all pairwise comparisons

**Fig. 3:**
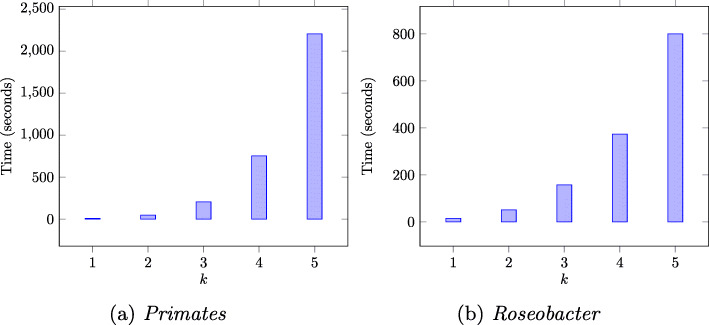
Run time to compute exact *ACS*_*k*_

It can also be observed in Figs. 1a and b that compared to both *kmacs* and our method, *ALFRED-G* has a much lower error percentage. However, in terms of runtime, our method runs 1.5–2.5X faster than that of *ALFRED-G* as illustrated by the runtime plots in Figs. 2a, b and c. As expected, the runtime grows approximately linearly as *k* increases. In constrast, the timings for the exact methods grows exponentially for both the *Primates* ranging from 8.7 seconds for *k*=1 to 2202.83 seconds for *k*=5. Similar behavior is observed for the *Roseobacter* dataset ranging from 15.04 seconds for *k*=1 to 800.11 seconds for *k*=5 (Fig. 3).

For the *Primates* dataset, we also ran the software *MissMax*, a heuristic alignment-free method for sequence comparisons developed in [21]. While the method produced an error rate of at most 0.52% with respect to exact values, its runtime is ≈145 – 185X slower than that of *ALFRED-G*.

For the *E. coli* full genomes dataset, the timings are shown in Fig. 2d. Note that the runtime is shown in hours as compared to seconds for the other datasets and only for *kmacs* and our method since *ALFRED-G* failed to complete its run in the allotted time of 72 hours.

To test the effectiveness of our approach for phylogeny construction in comparison to *kmacs* and *ALFRED-G*, we also constructed the phylogeny trees using our method as follows:

1. For every pair of input sequence in a dataset, compute *λk*′(·) both for X w.r.t. Y and for Y w.r.t. X.
2. Using approximate *ACS*_*k*_(X,Y) and *ACS*_*k*_(Y,X) estimated from *λk*′(·), compute the sequence similarity measure defined by Eq. 1.
3. Construct the symmetric distance matrix with entries filled with sequence similarity measures computed in the previous step. This matrix is of size 27×27,32×32 and 29×29 for *Primates*, *Roseobacter*, and *E. coli* datasets respectively.
4. Reconstruct phylogenetic tree using the neighbor program in the *PHYLIP* software suite [19] with the distance matrix as its input. The neighbor program constructs the phylogeny tree with Neighbor Joining methodology.
5. Compute the Robinson–Foulds distance (R-F) distance w.r.t the reference tree using the treedist program in the *PHYLIP* [19] software suite. Note that lower the R-F distance, better the matching of topology between two trees. If RF distance is zero, then there is an exact match between the two trees.
6. Repeat the above steps with *k*=1,…,10 for *Primates*, *Roseobacter* and *BAliBASE* datasets, and with *k*=1,…,7 for the *E. coli* dataset.

**Fig. 4:**
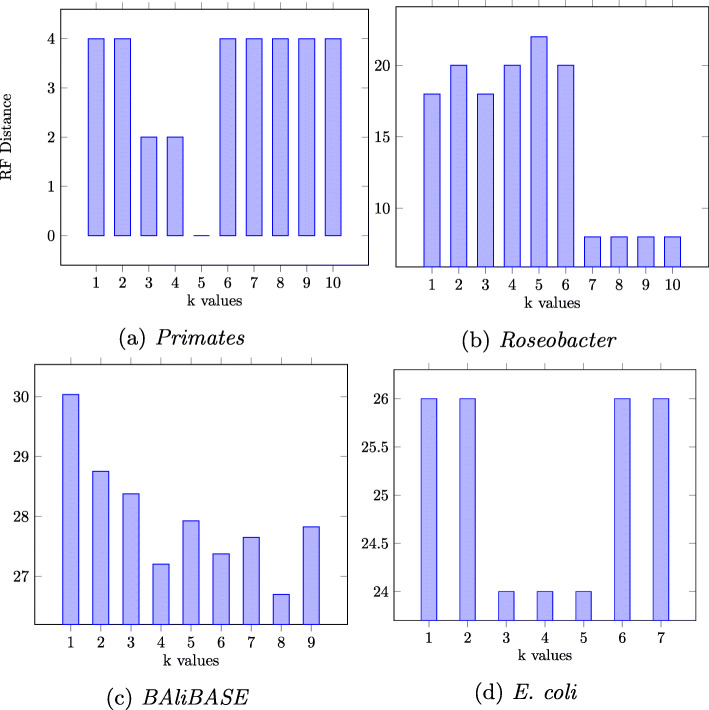
Robinson–Foulds distance with respect to the reference trees

For the *Primates* dataset, the Robinson–Foulds (RF) distance of the reconstructed tree with respect to the reference tree is 0 for *k*=5, 2–4 for other values of *k* (Fig. 4). For the same dataset *kmacs* reported an RF distance of 2–8, whereas *ALFRED-G* reported an RF distance of 0–2 [13]. Similar to *ALFRED-G*, our method was able to recover the expected phylogenetic tree for *Primates*. For the *Roseobacter* dataset, the RF distance of our algorithm are in the range of 20–8, and as the value of *k* is increases from 1 to 10, the RF distance tends to decline. *kmacs* and *ALFRED-G* reported RF distances, for the *Roseobacter* dataset, in the range of 18–10 and 18–8 respectively [13]. For *BAliBASE* datasets, all the three methods reported an average RF distances in the same range of 31–26. For *E. coli* dataset, both *kmacs* and our method reported RF distances in the same range of 24–26, while *ALFRED-G* was not able to complete within the alloted time limit of 24 hours per pair. Both *kmacs* and our method were able to complete their runs in less than 11 hours for *k*=5.

Finally, to evaluate the scalability of our algorithm in its ability to process genome-length sequences, we ran both *kmacs* and our method on the full genome sequences of 14 plant species. This dataset was originally compiled by [22] and is of total size 4.5 gigabases with the sequence lengths ranging from 111 megabases to 746 megabases. For the 50 pairs of sequences that both *kmacs* and our method were able to process in the allotted time limit of 72 hours per pair, our method was able to complete the runs in a average of 1.56 hours per pair, where as *kmacs* took an average of 4.67 hours per pair. This discrepancy in time is due to the difference in how *kmacs* and our method process the suffixes whose *LCP* is 0, which happens more often in longer genomes. Neither *ALFRED-G* nor the exact method is capable of processing smallest of the sequences of this dataset.

To summarize, our proposed method provides results more accurate than *kmacs* for the *Primates* and *Roseobacter* datasets, while being competitive in runtime compared to *kmacs* and much faster than *ALFRED-G*. In case of the *Primates* dataset, our method was able to recover the reference tree for *k*=5. For *BAliBASE* and *E. coli* datasets, the results are comparable to that of *kmacs*. With repsect to scalability, our method shows considerably improvement over that of *kmacs* for longer full genomes that are few hundred megabases long.

## Conclusions

In this paper, we presented a novel linear-time heuristic to compute the alignment-free measure of sequence similarity *ACS*_*k*_. We evaluate the accuracy of the *ACS*_*k*_ estimated from the proposed heuristic and demonstrated its applicability in construction of phylogeny trees.

We plan to extend this heuristic in the future in two different ways. Currently, all the published heuristics, including the one introduced in this work, can handle only mismatches and not insertions or deletions. We plan to adapt the proposed algorithm such that it allows insertions and deletions, where the key challenge is to manage is varying lengths of matched segments. Another way we plan to develop this heuristic is to enable forward and backward extensions on a 1-mismatch anchor segment.

## Data Availability

Datasets are available at http://alurulab.cc.gatech.edu/phyloand http://afproject.org/app/and the code is available at https://github.com/srirampc/adyar-rs

## Abbreviations

*ACS*: Average Common Substring
*ACS*_*k*_: Average Common Substring with *k* mismatches
UPGMA: Unweighted Pair Group Method for Phylogeny reconstruction
NJ: Neighbor-joining Method for Phylogeny reconstruction
*LCP*: Longest Common Prefix
*LCP*_*k*_: Longest Common Prefix while allowing *k* mismatches
*GST*: Generalized Suffix Tree
RMQ: Range miniumn query
